# Comparison of traditional field retting and *Phlebia radiata* Cel 26 retting of hemp fibres for fibre-reinforced composites

**DOI:** 10.1186/s13568-017-0355-8

**Published:** 2017-03-09

**Authors:** Ming Liu, Marcel T. Ale, Bartłomiej Kołaczkowski, Dinesh Fernando, Geoffrey Daniel, Anne S. Meyer, Anders Thygesen

**Affiliations:** 1 Center for Bioprocess Engineering, Department of Chemical and Biochemical Engineering, Technical University of Denmark, Søltofts Plads 229, 2800 Kongens Lyngby, Denmark; 2 Department of Forest Products/Wood Science, Swedish University of Agricultural Sciences, Vallvägen 9D, 750-07 Uppsala, Sweden

**Keywords:** Hemp fibre, Field retting, *Phlebia radiata* Cel 26, Microbial community, Enzyme profiling, Composite strength

## Abstract

Classical field retting and controlled fungal retting of hemp using *Phlebia radiata* Cel 26 (a mutant with low cellulose degrading ability) were compared with pure pectinase treatment with regard to mechanical properties of the produced fibre/epoxy composites. For field retting a classification of the microbial evolution (by gene sequencing) and enzyme profiles were conducted. By phylogenetic frequency mapping, different types of fungi, many belonging to the *Ascomycota* phylum were found on the fibres during the first 2 weeks of field retting, and thereafter, different types of bacteria, notably *Proteobacteria*, also proliferated on the field retted fibres. Extracts from field retted fibres exhibited high glucanase activities, while extracts from *P. radiata* Cel 26 retted fibres showed high polygalacturonase and laccase activities. As a result, fungal retting gave a significantly higher glucan content in the fibres than field retting (77 vs. 67%) and caused a higher removal of pectin as indicated by lower galacturonan content of fibres (1.6%) after fibres were retted for 20 days with *P. radiata* Cel 26 compared to a galacturonan content of 3.6% for field retted fibres. Effective fibre stiffness increased slightly after retting with *P. radiata* Cel 26 from 65 to 67 GPa, while it decreased after field retting to 52 GPa. Effective fibre strength could not be determined similarly due to variations in fibre fracture strain and fibre-matrix adhesion. A maximum composite strength with 50 vol% fibres of 307 MPa was obtained using *P. radiata* Cel 26 compared to 248 MPa with field retting.

## Introduction

The use of cellulosic fibres in high grade composites has gained increased interest over the last decade (Faruk et al. 2012; Liu et al. 2016b). Plant fibres originating from hemp (*Cannabis sativa* L.) are considered a particularly promising renewable raw material for production of high quality reinforcement of composite materials due to their high stiffness and strength to weight ratio (Faruk et al. 2012).

The cellulose-rich hemp fibres are present in the outermost bast layer of the hemp stem consisting of (a) epidermis, (b) several layers of primary fiber cells (cortex layer with large cells each containing a good-sized secondary wall), (c) layers of secondary cells that are smaller with a thinner secondary wall and (d) parenchyma cells. In the bast layer the primary and secondary fiber cells are bonded together by a pectinaceous matrix, the middle-lamella. The retting process is used to separate these cellulosic fibres by degrading the parenchyma cells, as well as in the middle lamella between fibres (Liu et al. 2015a, b). The removal of non-cellulosic components and separation of the cellulose fibers can also improve interface bonding between fibers and composite matrix polymers and thus increase the mechanical properties of the fiber reinforced composites (Li et al. 2009; Liu et al. 2016a, b).

During classic field retting in many European countries, hemp plants are cut and seeds collected with a specialized harvesting machine. Thereafter the plant stems are left on the field for a long period (20–70 days), processed into bales and finally separated into fibres and shives. The retting process is uncontrolled microbial wise, which can result in loss of fibre strength due to cellulase activity secreted by proliferation of native microorganisms on the hemp stems (Liu et al. 2015a). This process has been studied and involves fungi such as *Cladosporium* sp. and *Cryptococcus* sp. as well as bacteria including *Escherichia coli* (Brown and Sharma 1984; Ribeiro et al. 2015). The characteristics of the microorganisms involved in field retting, and their expressed enzymes should be studied to acquire a better understanding of the influence of field retting on chemical composition and mechanical properties of the fibres.

In order to avoid degradation of cellulose and reduction of fibre mechanical properties microbiologically controlled retting using selected fungi to degrade non-cellulosic components in the fibres has been investigated (Thygesen et al. 2007; Liu et al. 2015b). *Phlebia radiata* Cel 26 and *Ceriporiopsis subvermispora*, belonging to the *Basidiomycota* phylum, produce less cellulase enzymes compared to their wild types (Nyhlen and Nilsson 1987). *P. radiata* Cel 26 has been found most selective in pectin degradation resulting in higher fibre strength (Liu et al. 2015a). Nevertheless, the determination of enzyme activity in the extracts of *P. radiata* Cel 26 retted hemp fibres can provide knowledge on how the retting process can be optimized to produce high quality fibres.

It can thereby be hypothesized that higher cellulase activities would be detected in enzyme extracts from field retted hemp fibres compared to *P. radiata* Cel 26 retted fibres and that any higher cellulase activity in field retted samples would correlate to decreased mechanical properties. As a result, it can be expected that fibres with low mechanical properties should be obtained after field retting.

The objective of this study was to compare classical field retting with a pure microbial retting approach (*P. radiata* Cel 26) and a pure enzymatic retting approach (pectinase enzymes) based on mechanical properties of the obtained hemp fibre reinforced composites. Genetic identification and environmental scanning electron microscopy (ESEM) observation of bacteria and fungi was carried out to identify their abundance on hemp fibre surfaces. Enzymatic and microbial characteristics were linked to changes in chemical composition of fibres and mechanical properties of the fibres and fibre/epoxy composites. Finally the changes in microbial community, fibre composition and fibre mechanical properties were followed versus the retting duration. The correlation between microbial retting duration, chemical composition of fibres, and mechanical properties of produced composites was thereby established.

## Materials and methods

### Raw materials and fibre treatments

Hemp (*Cannabis sativa* L.), variety USO-31, was sown on May 5th 2013 [N 48.8526°, E 3.0190° (WGS84)] by Bafa Neu GmbH and harvested on Sep 6th 2013 (Liu et al. 2015a). Whole hemp plants were cut 5 cm above ground to obtain untreated bast fibres. Fibres representing the whole stems were applied in the study.

#### Field retting

The hemp was harvested with a Deutz-Fahr 6090 HTS leaving the chopped plant stems on the field. Field retting was conducted for 7, 14 and 20 days after harvest as previously reported (Liu et al. 2015a). After field retting, hemp stem samples for microbial evolution study and enzyme activity measurements were stored frozen until analysis, while for tensile test, chemical composition analysis and composite manufacturing, the samples were dried at 40^o^C directly after treatment.

#### Washing and autoclaving followed by fungal retting and pectinase treatment

Whole hemp stems were cut into 15 cm long pieces and washed three times at 40 °C for 5 min each using 500 mL water per 15 g stems. Fungal retting with *P. radiata* Cel 26 was carried out on hemp stem pieces after autoclaving the washed stem pieces at 121 °C for 60 min (HTT) to avoid contamination with wild fungi. The fungal retting was conducted for 7, 14 and 20 days in 1 L Erlenmeyer flasks (15 g hemp stems of 15 cm in length per flask) at 28 °C. *P. radiata* Cel 26 was obtained from the Swedish Agricultural University, Uppsala, Sweden (Nyhlen and Nilsson 1987; Liu et al. 2015b). After fungal retting, hemp stem samples for microbial evolution study and enzyme activity measurements were stored frozen, while for tensile test, chemical composition analysis and composite manufacturing, the samples were dried at 40^o^C directly after treatment.

Autoclave pretreatment of the washed hemp stems was both tested at 121 °C for 30 and 60 min. The following pectinase treatment was performed for 90 min on hand peeled hemp bast. This pectinase treatment was both tested with and without 30 min autoclave pretreatment (Liu et al. 2016b).

### Fungal and bacterial classification by gene sequencing

Hemp fibres were isolated manually from stems by removing xylem using a scalpel. Approximately 50 mg of sample (2 mm^2^) placed into 2 mL Eppendorf tubes was extracted directly for obtaining genomic DNA using PowerBiofilm™ DNA Isolation Kit (MO-BIO, Carlsbad, USA) according to the manufacturer’s instructions. Polymerase chain reaction (PCR) amplification for both bacterial and fungal DNA was carried out using a C1000™ thermo-cycler (BIO-RAD, Hercules, USA). Each DNA sample (1 μL) was used as template in the PCR reactions (Sun et al. 2015). The universal bacterial 16S ribosomal ribonucleic acid (16S rRNA) primers used were 27F (5′-AGAGTTTGATCATGGCTCA-3′) and 1492R (5′-CGGTTACCT TGTTACGACTT-3′). The fungal primer set was ITS5 (5′-GGAAGTAAAAGTCGTAACAAGG-3′) and ITS4 (5′-TCCTCCGCTTATTGATATGC-3′) (Eurofins Genomics, Ebersberg, Germany) (White et al. 1990; Gardes et al. 1991). The internally transcribed spacer (ITS) is the DNA situated between the small-subunit rRNA and the large-subunit rRNA genes in the chromosome or the correspondingly transcribed region in the polycistronic rRNA precursor transcript. Extracted DNA (1 μL) was added to a PCR master mix (49 μL) containing 0.5 μM of primers, Phusion HF buffer (F-518), 200 μM dNTPs and 0.5 U Phusion Hot Start II DNA polymerase (#F-549L; Thermo Fisher Scientific, Waltham, USA) (Ale et al. 2016).

PCR products were purified using GFX PCR DNA and Gel Band Purification Kit (GE28-9034-70 Sigma-Aldrich, Gillingham, UK). Cloning was performed using the pJet1.2/Blunt cloning vector (50 ng/µL) and T4 DNA ligase (5 U/µL). Ligation was carried out according to the manufacturer’s instructions (CloneJET PCR Cloning Kit #K1231, Thermo Scientific, USA) and the ligated product was used for transformation of electro-competent *E. coli* DH5α using BioRad Micropulser (BioRad, Hercules, USA). Purified plasmids were sequenced using the 27F primer for bacteria and the ITS4 primer for fungi synthesized by the company Macrogen Europe (Amsterdam, The Netherlands). The identified sequences are published in the EMBL Nucleotide Sequence Database with the accession numbers LT622055-LT622085 outlined in Tables 2 and 3 for bacteria and fungi, respectively.

A GenBank nucleotide database search was conducted using the BLAST algorithm (Basic Local Alignment Search Tool) to determine the closest relative of partial 16S gene sequences (Maidak et al. 1999). For each DNA sequence, a multiple alignment was created by Clustal W (Thompson et al. 1994). Evolutionary analysis and a phylogenic tree were constructed in Mega 6.0 with the Kimura two-parameter model (Kimura 1980). The reliability of the branches was evaluated with non-parametric bootstrapping (100 replicates). All positions with less than 95% site coverage were eliminated (complete deletion option). That means that fewer than 5% alignment gaps, missing data and ambiguous bases were allowed at any position.

### Protein extraction and enzyme activity assay

Hemp bast fibres were gently peeled from field retted and *P. radiata* Cel 26 retted hemp stems. For enzyme extraction, 4 g of bast fibres were submersed in 40 mL of 20 mM citrate buffer (pH 6.0) supplemented with 0.1% (v/v) Tween 20 and 0.25 mM dithiothreitol in a glass tube at 0 °C and shaken at 120 rpm for 1 h. For each sample, the crude enzyme extracts were concentrated until the volume was reduced to 3.0 mL (Suwannarangsee et al. 2014). After concentrating, the enzyme extracts were kept at 4 °C for activity assay. Protein content of enzyme extracts were determined using Bovine serum albumin as standard (Bradford 1976).

The pectinolytic, cellulolytic and hemicellulolytic enzyme activities were determined in crude enzyme extracts from the field retted and *P. radiata* Cel 26 retted samples at 40 °C in 20 mM citrate buffer (pH 6.0). Glucanase, polygalacturonase, galactanase and xyloglucan (XG)-specific endoglucanase activities were determined by measuring formation of reducing ends. The substrates used were for glucanase 10 g/L carboxymethyl cellulose (Suwannarangsee et al. 2014), for polygalacturonase 2 g/L polygalacturonic acid (Thomassen et al. 2011), for galactanase 5 g/L potato galactan (Michalak et al. 2012) and for xyloglucan 10 g/L tamarind xyloglucan (Benko et al. 2008). Enzyme to substrate ratio of 5:1 (v/v) was used for all the enzyme activity assays. The amount of reducing sugars that were liberated was measured by using 4-hydroxybenzoic acid hydrazide as colorimetric agent (Lever 1973). One unit of enzyme activity was defined as the volume of crude enzyme extracts (µL) required to liberate 1 µmol reducing ends (glucose equivalents) per minute under the assay conditions.

Laccase activity was measured by monitoring the oxidation of Diammonium 2,2′-azino-bis(3-ethylbenzothiazoline-6-sulfonate) (ABTS) by the enzyme extracts at 420 nm (Li et al. 2008). ABTS has the CAS Number: 30931-67-0 and was bought at Sigma-Aldrich, Gillingham, UK (Product no. 10102946001). *P. radiata* Cel 26 produces low levels of H_2_O_2_ and other peroxidases, which show activity with ABTS in the presence of H_2_O_2_ (Srinivasan et al. 1995). Therefore, catalase (Sigma-Aldrich, Gillingham, UK) was added at 1000 U/(mL of enzyme extracts) into crude enzyme extracts and incubated for 1 h at 37 °C to remove H_2_O_2_. One unit of laccase activity was defined as the volume of crude enzyme extract (µL) required to oxidize 1 µmol ABTS per minute under the assay conditions.

### Characterization of fibres

#### Chemical composition analysis

Dried bast fibres were ground with a microfine grinder (IKA, MF 10.1; IKA^®^-Werke GmbH, Staufen, Germany) through a 1-mm screen. Ground samples were extracted in a Soxhlet apparatus (Liu et al. 2015a) and the extractive-free fibres hydrolysed using a two-step sulfuric acid process (Sluiter et al. 2011). After acid hydrolysis, the hydrolysate was collected for monosaccharide analysis, and Klason lignin (i.e. residue of the hydrolysis) content was gravimetrically determined. The chemical composition of the hydrolysate was analysed by high-performance anion-exchange chromatography with pulsed amperometric detection (HPAEC-PAD) and with recovery values of the monosaccharides estimated from parallel runs (Arnous and Meyer 2008).

#### Scanning electron microscopy of fibre surface

Samples (5 mm long × 2 mm wide) were cut from bast fibre strips under a stereo microscope and fixed in 3% v/v glutaraldehyde, +2% paraformaldehyde in 0.1 M Na-cacodylate buffer (pH 7.2). After fixation, the samples were dehydrated in aqueous ethanol using: 20, 40, 60, 80, 90 and 100% for 15 min in each solution. Subsequent dehydration was performed in 33, 66 and 100% acetone in ethanol before samples were critical point dried using an Agar E3000 critical point dryer (Agar Scientific, Stansted, UK) with liquid CO_2_ as drying agent. Digital photos were taken using an XL 30 ESEM microscope (Philips, Eindhoven, Holland) operated at 10–15 kV (Fernando and Daniel 2008).

#### Tensile properties of fibres

Bast fibre strips (60 mm long × 1 mm wide) of mass in the range of 5–20 mg were used for tensile testing (Liu et al. 2015a). Tensile testing was performed using an Instron Testing Machine 2710-203 equipped with a 1kN load cell (Instron^®^, Buckinghamshire, United Kingdom). The gauge length was 10 mm and the displacement rate 0.5 mm/min (corresponding to a strain rate of 5% min^−1^). Tensile testing was performed on 20 specimens for each treatment. The cross-sectional area (A_f_) was determined from measured fibre mass, length and density [1.50 kg/dm^3^ (Cheung et al. 2009)]. Stiffness (linear regression in the strain interval 0.05–0.25%), ultimate tensile strength (UTS), and failure strain were determined based on measured stress–strain curves.

### Composite manufacturing and mechanical properties

#### Manufacturing of fibre/epoxy composites

Composites were manufactured by manually aligning the treated hemp bast fibre strips resulting in unidirectional composites. Epoxy resin (Araldite® LY 1568) and its amine hardener (Aradur® 3489) both supplied by Huntsman Corporation, Houston, USA, were mixed at a 100/28 mass ratio and degassed in a vacuum oven. The setup for vacuum infusion and mould processing has been described (Liu et al. 2016b). After demoulding, composite samples with dimensions of 140 mm × 10 mm × 2 mm were obtained and then glass fibre/epoxy tabs with lengths of 50 mm were mounted on composite specimens using epoxy adhesive (DP 460; 3M, Minneapolis, USA).

The volumetric composition of the composites was varied by varying the fibre weight contents (W_f_) in the range 0–0.70 g/g composite. When W_f_ was below 0.30, the composite specimens had irregular surfaces and their density (ρ_c_) was determined by the buoyancy method (Archimedes principle) using water as displacement medium. Otherwise the composite specimens had flat surfaces and ρ_c_ could be calculated based on their dimensions (i.e. length, width and thickness). The volumetric composition of composite samples was determined as previously described (Liu et al. 2016b). In the composites, the porosity (V_p_) was assumed to increase linearly versus the fibre volume content (V_f_) based on a proportionality constant (porosity factor) α_pf_ (Eq. 1). The porosity of hemp fibre reinforced composites can be separated into three main types: (a) fibre lumen; (b) voids among fibre bundles; and (c) voids at the interface between matrix polymer and fibre bundles (Liu et al. 2016a). The matrix correlated porosity factor (α_pm_) was assumed to be zero since no air bubbles were observed in the cured epoxy matrix by ESEM (Liu et al. 2016a).

1 $$V_{p} = \alpha_{pf} \times V_{f}$$

#### Tensile properties of composites

For tensile testing of the composite specimens, an Instron Testing Machine 5566 (Instron^®^, Buckinghamshire, United Kingdom) with a load cell of 10 kN was used. Strain measurements were conducted using two extensometers and the displacement rate was 1 mm/min (corresponding to a strain rate of 2.5% min^−1^). Based on measured stress–strain curves, composite stiffness (E_c_) (linear regression in the strain interval 0.05–0.25%) and UTS_c_ were determined. For each treatment, at least ten specimens with varied fibre content were tested. The UTS and stiffness curves of the composites were compared by assessing the responses by linear regression comparison within the tested range of V_f_. Effective fibre stiffness (E_f_) in the composites were thereby determined from Eq. 2 (Curtin 1993). The intercept was set equal to the measured matrix stiffness (E_m_).

2 $$\begin{aligned} E_{c} = E_{m} V_{m} + E_{f} V_{f} = E_{m} \left( {1 - V_{f} - V_{p} } \right) + E_{f} V_{f} \hfill \\ \Updownarrow \hfill \\ E_{c} = E_{m} \left( {1 - V_{f} - \alpha_{pf} V_{f} } \right) + E_{f} V_{f} = E_{m} + V_{f} \left( {E_{f} - E_{m} \left( {1 + \alpha_{pf} } \right)} \right) = E_{m} + kV_{f} \hfill \\ \Updownarrow \hfill \\ E_{f} = k + E_{m} \left( {1 + \alpha_{pf} } \right) \hfill \\ \end{aligned}$$

where the subscripts c, f and m indicate composite, fibres, and matrix, respectively and *k* is the slope of the regression line, i.e. of E_c_ vs. V_f_. The effective fibre strength cannot be calculated similarly due to effects of fibre-matrix adhesion and Weibull distribution of the fibre strength (Curtin 1993; Shah et al. 2016). It means that a part of the fibres are broken and not carrying load before the composite fails while some fibres still carry a reduced load due to their matrix adhesion. The composite strength was therefore calculated by linear regression for composites with 50 vol% fibres based on interpolation.

## Results

### Chemical composition of field retted and *P. radiata* Cel 26 retted fibres

Table 1 shows the changes in chemical composition of the hemp fibres after autoclaving, field retting and *P. radiata* Cel 26 retting. Before the retting with *P. radiata* Cel 26 it was required to autoclave pretreat the hemp stems for 60 min to avoid fungal contamination. The HTT treatment reduced the galacturonan content from 8.3 to 4.5% while no significant effects happened to the cellulose, hemicellulose and lignin contents (Liu et al. 2016b) (Table 1). In addition, the effects of 30 and 60 min HTT time appeared to be similar. A reduction in galacturonan, arabinan, and xylan contents were noted with increasing retting time during both treatments (Table 1). During field retting, the galacturonan content gradually decreased from 8.3% for untreated fibres, to 5.4% after 7 days and to 3.1% after 14 and 20 days. In contrast, the galacturonan content decreased to a lower level of only 1.6% galacturonan during 20 days retting with *P. radiata* Cel 26 partly due to the required HTT process. After 20 days arabinan and xylan contents decreased to 0.5 and 1%, respectively, both by field retting and by retting with *P. radiata* Cel 26. In comparison to untreated fibres, no increase in glucan content was noted during field retting. In contrast, the relative glucan content in the hemp fibres increased after only 7 days of retting with *P. radiata* Cel 26 from 67 to 82% (Table 1). The retting with *P. radiata* Cel 26 also resulted in higher glucan content than the pectinase treatment (69–71%) (Liu et al. 2016b).

**Table 1:** Chemical composition of hemp fibres after different treatments

Treatment	Treatment period	Amount (%)						
		Glu	GalA	Gal	Ara	Xyl	Man	Lignin
Untreated	0	60.0 (2.2)^f^	7.3 (0.4)^a^	2.1 (0.2)	1.3 (0.3)	1.2 (0.1)	3.0 (0.3)	4.4 (0.3)^d^
Washed 40 °C	60 min	67.2 (2.6)^de^	8.3 (0.4)^a^	2.1 (0.1)	1.2 (0.1)	1.3 (0.2)	4.6 (0.2)	5.3 (0.2)^bcd^
HTT	30 min	67.0 (1.3)^de^	4.5 (0.4)^b^	1.8 (0.1)	0.8 (0.1)	1.1 (0.1)	4.5 (0.2)	4.6 (0.2)^cd^
HTT	60 min	67.9 (2.8)^de^	5.5 (0.8)^cd^	2.1 (0.3)	1.1 (0.3)	1.4 (0.2)	3.5 (0.4)	5.0 (0.3)^cd^
Field retting	7 days	63.9 (1.0)^ef^	5.4 (0.6)^bc^	2.0 (0.2)	0.7 (0.2)	1.1 (0.2)	4.0 (0.4)	5.3 (0.9)^bcd^
	14 days	70.2 (0.5)^cde^	3.1 (0.4)^efg^	2.4 (0.2)	0.4 (0.0)	0.8 (0.2)	3.4 (0.0)	6.1 (0.9)^bc^
	20 days	66.9 (1.2)^de^	3.6 (0.4)^def^	2.0 (0.0)	0.6 (0.1)	1.0 (0.2)	3.9 (0.2)	8.1 (0.3)^a^
Fungal retting	7 days	81.8 (1.6)^ab^	4.2 (0.2)^de^	2.4 (0.1)	0.7 (0.0)	1.3 (0.1)	4.7 (0.3)	6.1 (1.1)^bc^
	14 days	83.4 (3.8)^a^	2.0 (0.2)^gh^	2.3 (0.2)	0.5 (0.1)	1.3 (0.1)	4.4 (0.3)	5.3 (0.1)^bcd^
	20 days	76.7 (1.5)^bc^	1.6 (0.0)^h^	2.0 (0.0)	0.5 (0.0)	1.1 (0.0)	4.5 (0.4)	6.7 (0.5)^ab^
Pectinase^A^	90 min	71.8 (0.8)^cd^	4.3 (0.2)^cd^	1.7 (0.0)	0.7 (0.0)	0.9 (0.0)	5.6 (0.1)	4.8 (0.6)^cd^
HTT + pectinase^A^	90 min	69.5 (3.9)^de^	2.9 (0.3)^fg^	1.5 (0.1)	0.7 (0.1)	0.7 (0.1)	4.2 (0.3)	4.2 (0.5)^d^

Values are means (standard deviation) for three replicates. In each column, values that do not share a letter are significantly different at the 5% level

*Glu* glucan, *GalA* galacturonan, *Gal* galactan, *Ara* arabinan, *Xyl* xylan, *Man* mannan, *Lignin* Klason lignin

^A^With or without autoclave treatment (HTT) at 1 bar (121 °C) for 30 min before the pectinase treatment (Liu et al. 2016b)

Besides these changes, a slight increase in lignin content (i.e. lignin to carbohydrate ratio), particularly during field retting was noted presumably due to a lack of lignin degrading enzymes produced by the microbial retting flora. The increase can also be due to microbial biomass formation and that the degradation rate of lignin is much lower than that of other components i.e. pectin.

### Evolution of the microbial community during field retting

**Fig. 1:**
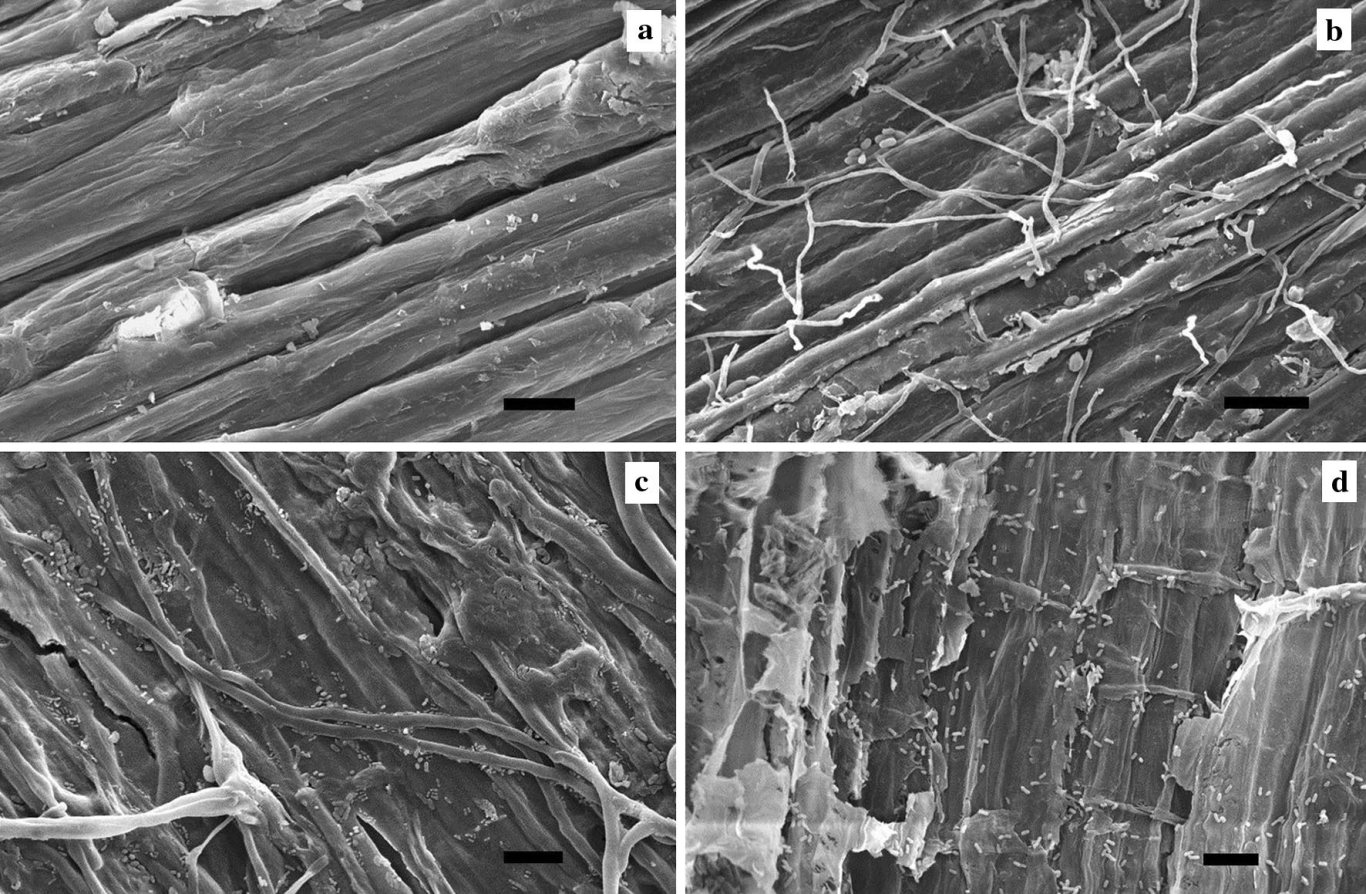
ESEM microscopy images showing presence of bacteria and fungi in field retted hemp after 0 (**a**), 7 (**b**), 14 (**c**) and 20 days (**d**). *Scale bars* **a**, **b**, 20 µm; **c**, **d**, 10 µm

**Fig. 2:**
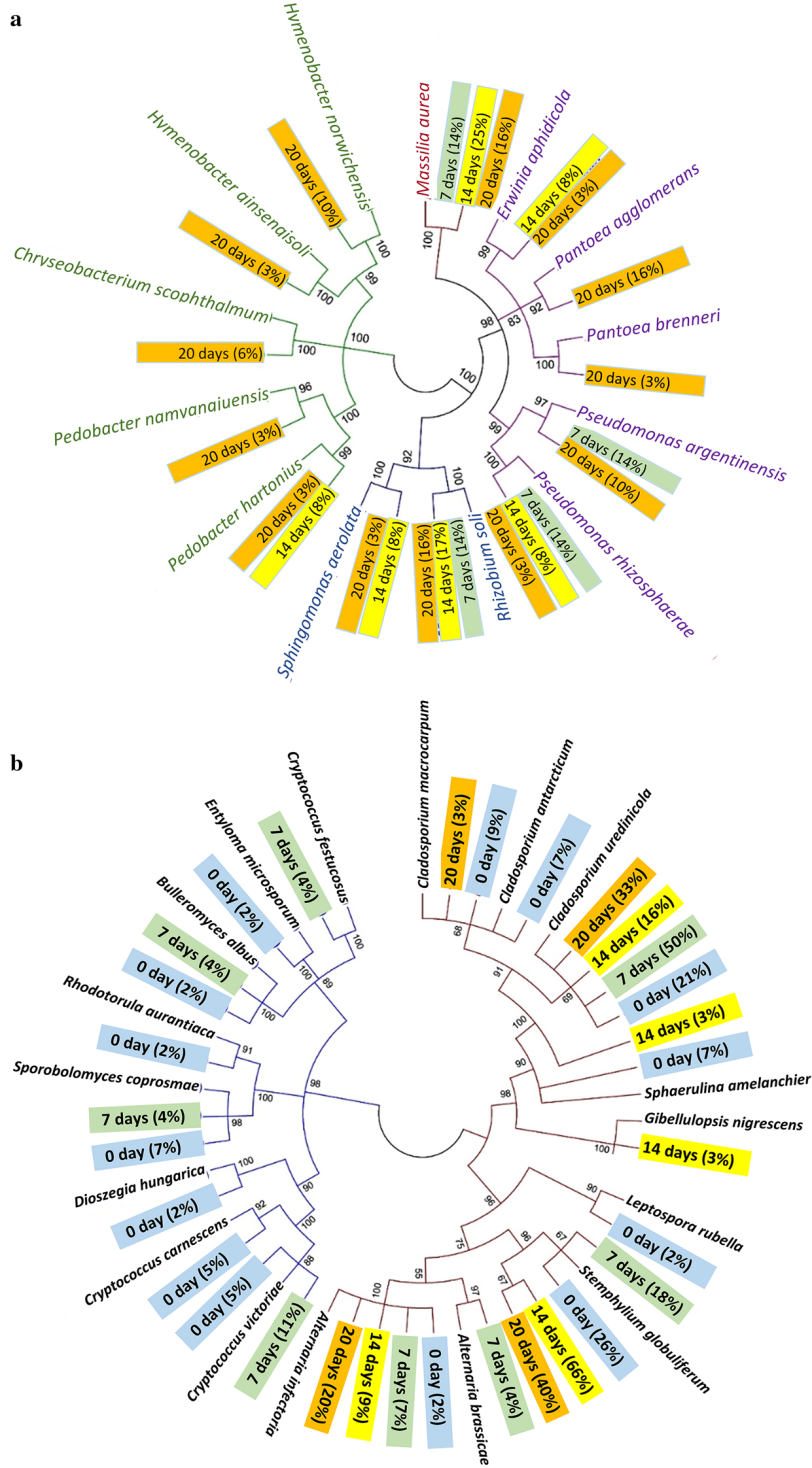
Phylogenetic tree of bacterial community (**a**) and fungal community (**b**) present in the hemp fibre samples. The *numbers* in the figures show the bootstrap values between the branches. In **a** *α*-, *ß*-, and *γ*-*proteobacteria* have *blue*, *brown* and *purple* braches, respectively, while *Bacteroidetes* have *green* branches. In **b** *Ascomycota* and *Basidiomycota* fungi have *brown* and *blue* branches, respectively. Relative abundance is shown in % calculated based on the number of bacterial (**a**) and fungal (**b**) clones at each retting time. Samples are sorted according to retting time (0 days = *blue*; 7 days = *green*; 14 days = *yellow*; 20 days = *orange*)

The changes in chemical composition of fibres during field retting (Table 1) were accompanied by changes in the microbial community versus time. Figure 1 shows typical examples of the proliferation and pervasive action of fungi and bacteria on hemp fibre surfaces. In addition, gene sequencing provided identification and diversity of bacteria and fungi as shown in Tables 2 and 3, respectively. Phylogenetic trees of the bacterial and fungal community evolution during field retting are shown in Fig. 2a, b, respectively.

**Table 2:** Phylogenetic frequency and affiliation of bacteria on hemp fibres retted for different times

Phylum	Bacterial species		% of total bacterial community^a^ Field retting time (day)				Accession number
	Genus	Species					Old (similarity-%) new
			0	7	14	20	
*α*-*Proteo*-*bacteria*	*Rhizobium*	*soli*	0	14 [1]	17 [2]	16 [5]	NR115996 (97%) LT622055
	*Sphingomonas*	*aerolata*	0	0	8 [1]	3 [1]	NR042130 (98%) LT622056
*ß*-*Proteo*-*bacteria*	*Massilia*	*aurea*	0	14 [1]	25 [3]	16 [5]	NR042502 (99%) LT622057
*γ*-*Proteobacteria*	*Erwinia*	*aphidicola*	0	0	8 [1]	3 [1]	NR104724 (99%) LT622058
	*Pantoea*	*agglomerans brenneri*	0	0	0	16 [5]	NR041978 (99%) LT622059
			0	0	0	3 [1]	NR116748 (99%) LT622060
	*Pseudomonas*	*argentinensis* *rhizosphaera* *syringae*	0	14 [1]	0	10 [3]	NR043115 (97% LT622061
			0	14 [1]	8 [1]	3 [1]	NR029063 (99%) LT622062
			0	0	8 [1]	3 [1]	NR074597 (99%) LT622063
	*Shigella*	*sonnei*	0	29 [2]	8 [1]	0	NR074894 (96%) LT622064
*Bacteroidetes*	*Chryseobacterium*	*scophthalmum*	0	0	0	6 [2]	NR025386 (97%) LT622065
	*Hymenobacter*	*ginsengisoli* *norwichensis*	0	0	0	3 [1]	NR108904 (96%) LT622066
			0	0	0	10 [3]	NR042172 (99%) LT622067
	*Pedobacter*	*hartonius* *namyangjuensis*	0	0	8 [1]	3 [1]	NR104917 (95%) LT622068
			0	0	0	3 [1]	NR113980 (97%) LT622069
Totals % [no. clones]			0 [0]	100 [7]	100 [12]	100 [31]	

^a^Percentage based on total number of sequences for each retting period. In brackets is the number of clones identified

**Table 3:** Phylogenetic frequency and affiliation of fungi on hemp fibres retted for different times

Phylum	Fungal species		% of fungal community^a^ Field retting time (day)				Accession number
	Genus	Species					Old (similarity-%) new
			0	7	14	20	
*Ascomycota*	*Alternaria*	*brassicae*	0 [0]	4 [1]	0 [0]	3 [1]	KJ728680 (100%) LT622070
		*infectoria*	2 [1]	7 [2]	9 [3]	20 [6]	KC254057 (93%) LT622071
	*Cladosporium*	*antarcticum*	7 [3]	0 [0]	0 [0]	0 [0]	NR121332 (100%) LT622072
		*macrocarpum*	9 [4]	0 [0]	0 [0]	3 [1]	KC311478 (100%) T622073
		*uredinicola*	21 [9]	50 [14]	16 [5]	33 [10]	KP216999 (100%) LT622074
	*Gibellulopsis*	*nigrescens*	0 [0]	0 [0]	3 [1]	0 [0]	HE972037 (99%) LT622075
	*Leptospora*	*rubella*	2 [1]	0 [0]	0 [0]	0 [0]	HE774478 (95%) LT622076
	*Stemphylium*	*globuliferum*	26 [11]	18 [5]	66 [21]	40 [12]	KF479193 (99%) LT622077
*Basidiomycota*	*Bulleromyces*	*albus*	2 [1]	4 [1]	0 [0]	0 [0]	KC455879 (99%) LT622078
	*Cryptococcus*	*carnescens*	5 [2]	0 [0]	0 [0]	0 [0]	JX188120 (100%) LT622079
		*festucosus*	0 [0]	4 [1]	0 [0]	0 [0]	FR717832 (98%) LT622080
		*victoriae*	5 [2]	11 [3]	0 [0]	0 [0]	KM376379 (98%) LT622081
	*Dioszegia*	*hungarica*	2 [1]	0 [0]	0 [0]	0 [0]	EU286794 (99%) LT622082
	*Entyloma*	*microsporum*	2 [1]	0 [0]	0 [0]	0 [0]	AY081045 (94%) LT622083
	*Rhodotorula*	*aurantiaca*	2 [1]	0 [0]	0 [0]	0 [0]	AB093528 (99%) LT622084
	*Sporobolomyces*	*coprosmae*	7 [3]	4 [1]	0 [0]	0 [0]	KJ701199 (100%) LT622085
Totals % [no. clones]			100 [43]	100 [28]	100 [32]	100 [30]	

^a^Percentage based on total number of sequences for each retting period. In brackets is the number of clones identified

ESEM microscopy observation showed that neither bacteria nor fungal hyphae were present on the hemp fibre surface before field retting (Fig. 1a). After 7 days of field retting fungal hyphae were abundant but very few bacteria were observed (Fig. 1b). After 14 days, fungi were present on the hemp fibre surfaces as on fibres retted for 7 days, and bacterial proliferation was also evident and sometimes observed associated with fungal hyphae (Fig. 1c). In addition, spots of local decay on the fibre surface were observed. After 20 days, a higher bacterial population was observed all over the fibre surfaces while fungal hyphae abundance was decreased (Fig. 1d). Severe degradation of the fibre surface was observed at the locations with high bacterial colonization.

#### Bacterial community

16S rRNA from samples with different field retting durations was analyzed for bacterial diversity resulting in a total of 81 different sequences (Table 2). Sequences were grouped according to phylum affiliation including *α*-*ß*-*γ*-*proteobacteria* and *Bacteroidetes* (Table 2). In the unretted sample, no bacteria were found, but the sequencing identified chloroplast originating from the epidermis part of the plant stem (Vergara et al. 2016). From visual inspection, the green colour of chloroplast of the stems disappeared later. After 7 days retting, 14, 14, and 57% of the bacterial diversity belonged to *α*-, *ß*-, and *γ*-*proteobacteria*, respectively. After 20-days retting, the frequency percentages of *α*- and *ß*-*Proteobacteria* were similar (19 and 16% of the bacteria) and that of *γ*-*proteobacteria* decreased to 38%. *Bacteroidetes* were identified after 14 and 20 days of retting with 8 and 25% of the bacteria, respectively.

The most frequent phylotype was *Massilia aurea* after 7 and 14 days retting with frequencies of 14 and 25%, respectively, decreasing to 16% after 20 days. *Rhizobium soli* constituted 14–17% of the bacterial diversity during the whole retting period. The *Pseudomonas* fraction of the bacteria (including the species *argentinensis*, *rhizosphaera* and *syringae*) decreased from 28% after 7 days to 16% after 14–20 days. The *Shigella sonnei* part of the bacteria decreased from 29 to 0% after 20 days. In addition minor but increasing percentages (0 → 3–10% of bacteria) were found for *Erwinia aphidicola*, *Chryseobacterium scophthalmum*, *Hymenobacter* sp. and *Pedobacter* sp.

Figure 2a shows the genetic differences between the identified bacteria. It shows that few bacterial specie were present initially (7 days) and the evolution of a more varied bacterial community after 14 days with six yellow labels and an even more diverse community after 20 days with 13 orange labels.

#### Fungal community

Sequences of the fungal community derived from the ITS gene corresponded to five genera distributed within the *Basidiomycota* phylum and to six genera within the *Ascomycota* phylum (Table 3). Between 0 and 7 days of retting, the fungal community was dominated by *Ascomycota* contributing 74% of the diversity increasing to 100% after 14 days of retting. *Stemphylium globuliferum* was the most frequent species with 18–66% of the fungal community. Increases in frequency percentages were both observed for *Cladosporium uredinicola* (16 → 33%) and *Alternaria infectoria* (9 → 20%). However *C. uredinicola* dominated initially contributing 50% of the diversity in the fungal community after 7 days. Low and decreasing frequency percentages of *Cryptococcus* (including the species *carnescens*, *festucosus* and *victoriae*) and *Sphaerulina amelanchier* were observed with 10 and 7% of the fungal community after 0 days, respectively.

Figure 2b shows the genetic differences between the identified fungi and the extinction of *Basidiomycota* after 14 days of retting, presumably as a result of faster growth of *Ascomycota* fungi. The analysis also indicates that many well-known fungal species were present initially with 14 blue labels showing a high biodiversity. With time, the diversity of the fungal community decreased as evident from the decrease to only four orange labels after 20 days represented by *Alternaria*, *Stemphylium* and *Cladosporium* (Fig. 2b). This trend was opposite to the increasing diversity of the bacterial community during retting as illustrated in Fig. 2a.

### Enzyme activity changes during field—and fungal retting

**Fig. 3:**
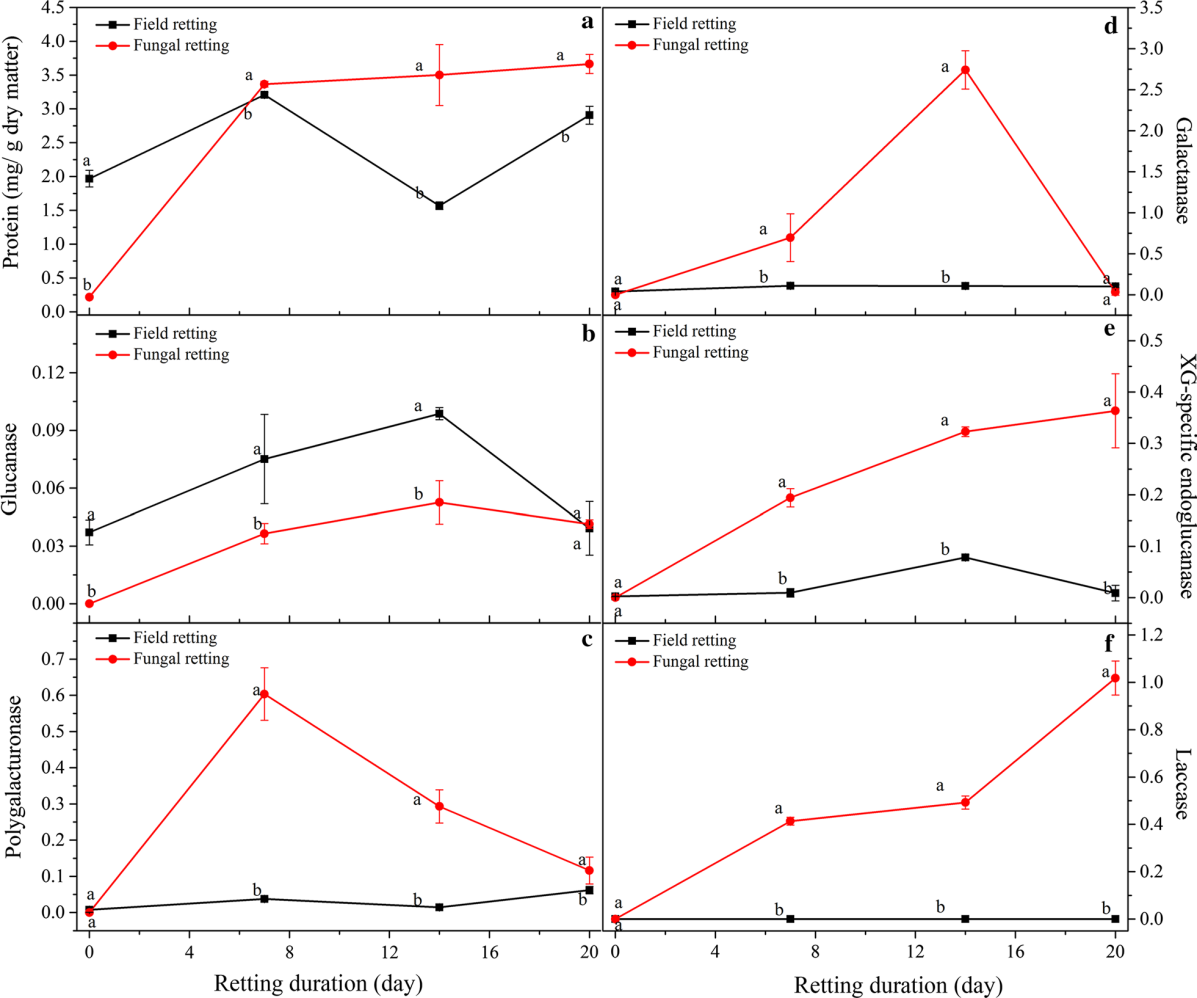
Protein content of enzyme extracts (**a**), and enzyme activities of glucanase (**b**), polygalacturonase (**c**), galactanase (**d**), XG-specific endoglucanase (**e**), and laccase (**f**) versus retting duration (Units of enzyme activities were shown as U/g dry of matter hemp fibres)

Figure 3 shows protein content and enzyme activities of the enzyme crude extracts from field retted and *P. radiata* Cel 26 retted samples at varied retting durations. As shown in Fig. 3a, enzyme extracts from *P. radiata* Cel 26 retted fibres had higher protein content compared to that from field retted fibres after 14–20 days. Comparison of different enzyme activities in extracts from field—and *P. radiata* Cel 26 retted fibres, showed that field retted fibres had significantly higher glucanase activity (0.03 U/g fibres) up to 20 days (Fig. 3b). High glucanase present in the extracts from field retted fibres corroborated with the chemical composition data (Table 1: low cellulose content) and microbial community evolution during field retting (Tables 2, 3: cellulose degrading microorganisms).

Much higher polygalacturonase activity in the extracts from *P. radiata* Cel 26 retted fibres of 0.6 U/g fibres was observed after 7 days compared to that extracted from field retted fibres (0.05 U/g fibres). Thereby the more efficient pectin degradation by fungal retting (Table 1) can be corroborated by the high polygalacturonase activity present in the enzyme extracts from *P. radiata* Cel 26 retted fibres (Fig. 3c). The polygalacturonase activity obtained with *P. radiata* Cel 26 gradually decreased to 0.1 U/g DM after 20 days.

Besides glucanase and polygalacturonase activities, galactanase, XG-specific endoglucanase, and laccase activities were determined for both field retted and *P. radiata* Cel 26 retted fibres. *P. radiata* Cel 26 retted fibres exhibited higher galactanase and XG-specific endo-glucanase activity than field retted fibres. Laccase activity was found to increase with extended *P. radiata* Cel 26 retting duration from 0.4 U/g DM after 7 days to 1.0 U/g DM after 20 days, while no laccase activity was detected in the crude enzyme extracts from field retted fibres (Fig. 3f).

### Mechanical properties of fibres and fibre/epoxy composites

#### Fibre bundle strength

The stiffness and UTS of the fibres are shown in Table 4 while the composite derived data are shown in Table 5. The untreated hemp fibres had stiffness of 29 GPa and UTS of 770 MPa. A decrease in fibre strength was observed with increased field retting duration to 683 MPa after 20 days (Table 4). The decrease in fibre strength can be explained by the loss of cellulose because of cellulase activities (Fig. 3b). For *P. radiata* Cel 26 retted fibres, the stiffness increased and UTS decreased slightly. After fibres were retted with *P. radiata* Cel 26 for 14 days, the stiffness and UTS of fibres were 42 GPa and 720 MPa. Only slight changes were observed on strength and stiffness by 30 and 60 min of HTT pretreatment. However HTT treatment appeared to increase fibre stiffness and strength slightly when followed by pectinase treatment.

**Table 4:** Mechanical properties of hemp fibres after different treatments

Treatment	Period	Stiffness (GPa)	UTS (MPa)	Strain (%)
Untreated	0	29 (3)^cd^	772 (104)^ab^	5.0 (0.9)^a^
HTT^A^	30 min	31 (6)^cd^	688 (120)^bc^	2.9 (0.7)^de^
HTT	60 min	32 (5)^bc^	785 (180)^ab^	2.8 (0.7)^de^
Field retting	7 days	33 (5)^bc^	832 (198)^a^	4.7 (1.8)^a^
	14 days	31 (5)^cd^	697 (104)^bc^	4.5 (1.5)^ab^
	20 days	28 (5)^cd^	683 (107)^bc^	4.5 (1.2)^ab^
Fungal retting	7 days	27 (5)^d^	707 (130)^a^	3.6 (0.9)^bcd^
	14 days	42 (5)^a^	720 (150)^abc^	2.3 (0.5)^e^
	20 days	31 (7)^cd^	714 (145)^bc^	3.1 (0.8)^cde^
Pectinase^A^	90 min	18 (4)^e^	636 (61)^c^	3.8 (0.9)^bc^
HTT + pectinase^A^	90 min	36 (5)^b^	777 (117)^ab^	2.6 (0.9)^e^

Values are shown as mean (standard error). In each column, values that do not share a letter are significantly different at the 5% level

^A^With or without autoclave treatment (HTT) at 1 bar (121 °C) for 30 min before the pectinase treatment (Liu et al. 2016b)

**Table 5:** Mechanical properties of hemp fibre composites after the different treatments

Treatment	Period	Composite properties (V_f_ = 50%)			Calculated fibre properties	
		Stiffness E_c_ (GPa)	UTS_c_ (MPa)	Porosity V_p_ (%)	E_f_ (GPa)	Porosity α_pf_ (%)
Untreated	0	33.8 (0.6)	294 (14)	7.9 (0.4)	64.9 (1.2)	15.7 (0.8)
Field retted	20 days	26.9 (0.6)	248 (17)	8.1 (0.6)	51.2 (1.1)	16.2 (1.2)
Fungal retting	7 days	32.2 (0.5)	283 (8)	6.6 (0.8)	61.6 (0.9)	13.1 (1.5)
	14 days	34.5 (0.5)	300 (9)	4.9 (0.4)	66.2 (0.9)	9.7 (0.7)
	20 days	34.8 (0.3)	307 (6)	5.5 (0.5)	66.9 (0.6)	10.9 (0.9)
Pectinase^A^	90 min	35.2 (1.0)	306 (15)	6.0 (0.3)	67.6 (1.9)	11.9 (0.6)
HTT + pectinase^A^	90 min	38.2 (0.8)	325 (6)	4.2 (0.2)	73.6 (1.6)	8.4 (0.4)

Values are means (standard error)

^A^With or without autoclave treatment (HTT) at 1 bar (121 °C) for 30 min before the pectinase treatment (Liu et al. 2016b)

#### Fibre reinforced composite results

**Fig. 4:**
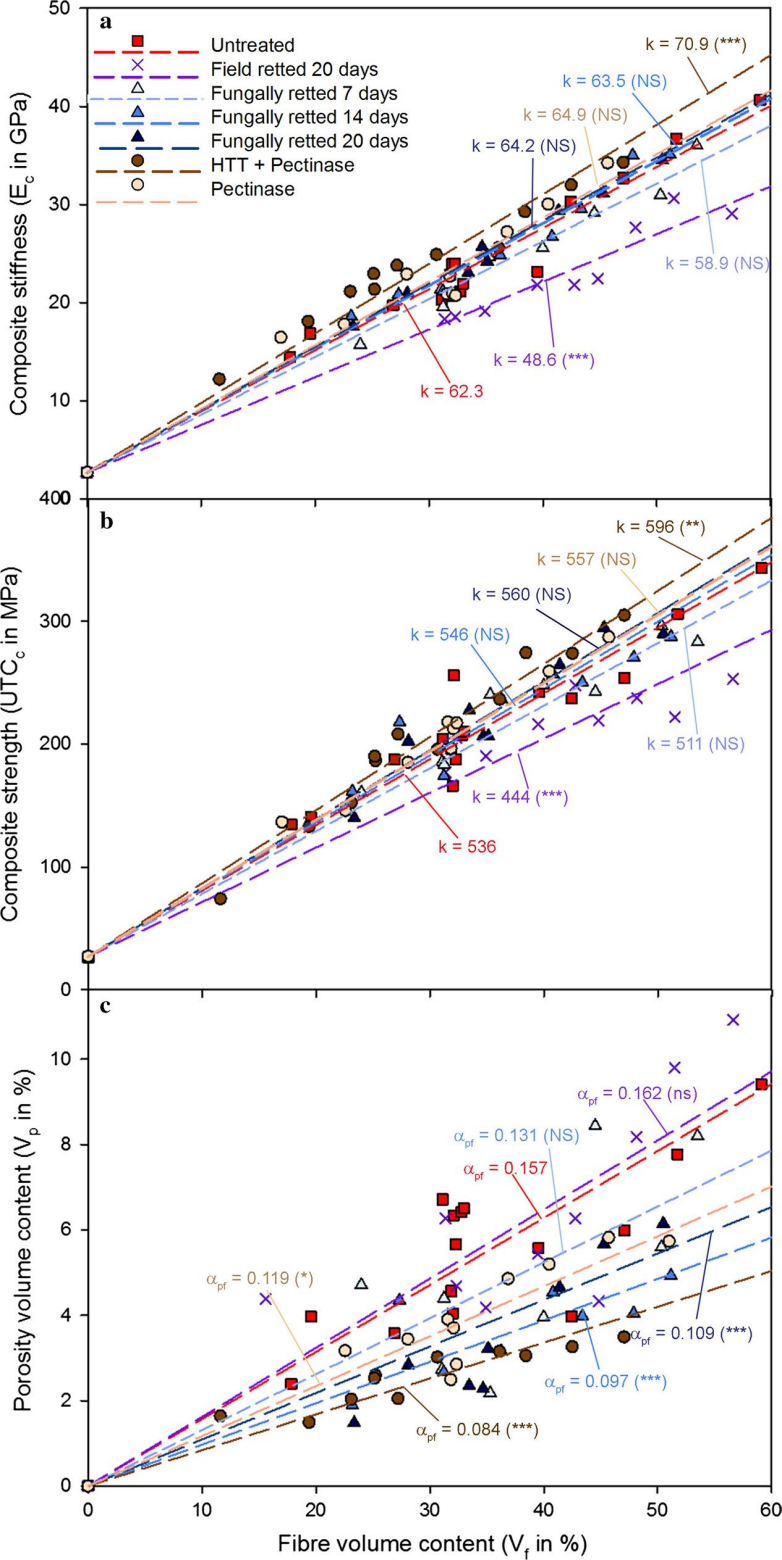
Stiffness (**a**), UTS (**b**) and porosity (**c**) of composites reinforced with untreated and treated fibres versus fibre volume (V_f_) contents (*k* is slope of the linear regression model lines). Pectinase treatment was performed with and without prior autoclave treatment (HTT) at 1 bar (121 °C) for 30 min (Liu et al. 2016b)

All fungal retted and untreated fibres were assessed in hemp fibre/epoxy composites. In order to compare with traditional field retting, the 20-day field retted fibres were also assessed. As shown in Fig. 4a, the starting points of the linear regression lines at V_f_ = 0, showed the measured epoxy matrix stiffness (E_m_) of 2.7 GPa. By comparison of the slopes (*k*) of the lines of E_c_ versus V_f_, of composites with differently treated fibres, it can be seen that composites with *P. radiata* Cel 26 retted fibres, irrespective of retting duration, had much higher stiffness and strength than with field retted fibres (Fig. 4). The composite stiffness and strength at V_f_ = 50% were determined and shown in Table 5. The composite strength and stiffness were retained by the retting with *P. radiata* Cel 26 for 7–20 days with 32–35 GPa and 283–307 MPa, respectively. This treatment effect is thereby similar to pectinase treatment and better than field retting giving only 306 and 248 MPa, respectively.

The effective fibre stiffness (E_f_) and strength (UTS_f_) were estimated by linear regression within the V_f_ range tested (Table 5). It was confirmed by correlation analysis that there was a linear increase in the tested V_f_ range with at least 99% significance. The untreated hemp fibres had E_f_ of 65 GPa. E_f_ was determined to be similar (62, 66 and 67 GPa) for 7-, 14-, and 20-days retted samples using *P. radiata* Cel 26, respectively. In contrast, 20 days field retted fibres showed that E_f_ was only 51 GPa. If *P. radiata* Cel 26 retting was replaced with pectinase treatment higher stiffness of 74 GPa was obtained. The results demonstrate thereby that *P. radiata* Cel 26 retting produced better fibres than field retting.

The porosity factors (α_pf_) of composites with differently treated fibres were determined by Eq. (1) based on the experimental data of V_p_ versus V_f_. In general, V_p_ increased versus V_f_ due to voids corresponding to cell lumen and unimpregnated void space between fibre bundles. As shown in Fig. 4c, composites with field retted and untreated fibres had the highest porosity factor (up to 0.16), followed by composites with *P. radiata* Cel 26 retted fibres (0.10–0.13). For composites made with *P. radiata* Cel 26 retted fibres, the composite porosity was found to decrease vs. the retting duration due to smaller fibre bundles in general obtained with more defibration (Thygesen et al. 2007; Liu et al. 2016a).

## Discussion

### Microbial community and enzyme expression

Field retting based on natural microbial communities and *P. radiata* Cel 26 retting was assessed in hemp fibre treatment for fibre/epoxy composites and compared with a pure enzymatic approach. The changes in chemical composition of the hemp fibres was consistent with a previous study where field retting caused greater loss of cellulose with increased duration (Liu et al. 2015a), while fungal retting with *P. radiata* Cel 26 did not degrade the cellulose (Liu et al. 2015b). It is a strong indication for cellulase production recorded during field retting (Fig. 3b) due to the presence of wild microbial populations. Normally with these types of wild microbial populations, the bacteria associate with the fungal hyphae so that they can get access to carbohydrates released by the fungal attack of the plant cell wall (Boonchan et al. 2000). Therefore the abundance of fungi decreased with extended retting duration, while the abundance and diversity of bacteria increased as observed with ESEM (Figs. 1, 2).

γ-*Proteobacteria* as the most abundant phylum in the bacterial community has also been observed with water retting of jute (*Corchorus olitorius*) (Munshi and Chattoo 2008). *γ*-*Proteobacteria* can hydrolyze cellulose and pectin owing to its cellulolytic and pectinolytic activity (Hrynkiewicz et al. 2010). *Rhizobium* sp. and *Massilia* sp. have also been found to produce cellulase enzymes (Morales et al. 1984; Hrynkiewicz et al. 2010), which explains the cell wall degradation at the bacterially colonized sites at late retting stages. *Pseudomonas* sp. were found to be particularly important in the decomposition of pectin in plant fibres during retting under aerobic conditions (Betrabet and Bhat 1958; Rosemberg 1965).

Endophytic fungi such as *S. globuliferum* and *Cladosporium* sp. can penetrate plant tissue while producing pectinase and cellulase enzymes (Brown and Sharma 1984; Wang and Dai 2011). *A. infectoria* has also been reported to perform enzymatic hydrolysis of cellulose and hemicellulose (Silva et al. 2014). This confirms that pectin and carbohydrate degrading enzymes were produced during field retting giving decay in the hemp fibre cell walls (Table 1) and agrees with the measured enzyme activities (Fig. 3). *Cryptococcus* can produce cutinase enzymes (Masaki et al. 2005) involved in cutin degradation in the cuticle on hemp stem surface, which explains why it was present initially. The zero laccase activity is consistent with the lack of *Basidiomycota* fungi in field retting. The higher Klason lignin content of the field retted fibres (Table 1) is presumably due to the zero laccase activity (Fig. 3, and consistent with lack of ligninolytic *Basidiomycota* fungi (Table 3).

### Fibre compositional changes and resulting composite properties

**Fig. 5:**
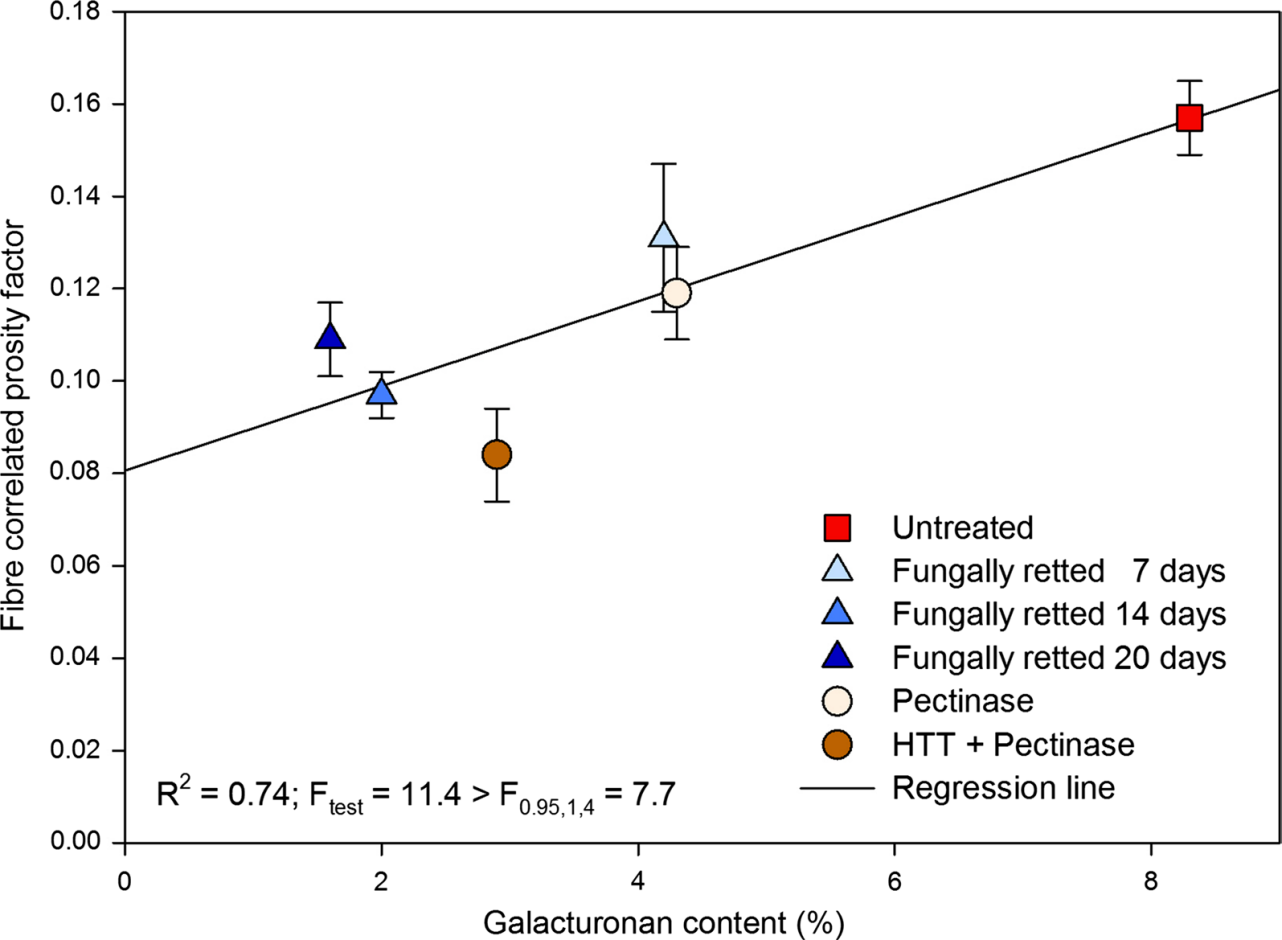
Correlation between fibre correlated porosity factor (α_pf_) and galacturonan content of fibres after different treatments

Mechanical properties of composites depend not only on fibre strength but also on the hemp fibre—matrix interface adhesion (Gassan et al. 2000). Improved interface adhesion with the fibres can be obtained with a low fibre surface porosity content (Li et al. 2009; Liu et al. 2016a). The decrease in composite porosity obtained with *P. radiata* Cel 26 and by pectinase treatment (Fig. 4c) were primarily due to degradation of parenchyma cells mainly consisting of pectin and hemicellulose. The fibre correlated porosity factor increased thereby versus the galacturonan content after retting with *P. radiata* Cel 26 similar with pectinase treatment (Fig. 5). The decrease in composite porosity could thereby explain the slightly better mechanical properties of the composites obtained with the *P. radiata* Cel 26 retted fibres (Fig. 4). However the recorded cellulase activity (Fig. 3b) might have reduced the resulting composite strength and stiffness slightly compared to pectinase treatment (Fig. 4a, b).

The fibre stiffness was two times higher when back calculated based on composite data (Tables 4, 5). The high fibre stiffness based on composite data can be explained due to the approximate half failure strain obtained of 1% in composites. The back calculation for calculation of fibre stiffness has proven to be possible since linear relationships are obtained (Shah et al. 2016). Technical fibres were in this study tested instead of single fibres since cross sectional area is more easily determined and due to bias in the isolation of single fibres from bundles by hand (Charlet et al. 2010).

### Perspectives for industrial implementation of fungal defibration

Comparing untreated hemp and fungal retted hemp slight increases were obtained in fibre stiffness from 65 to 67 MPa and in composite strength (Table 5) while the porosity factor decreased from 0.157 to 0.109. The obtained decrease in porosity content results in a more homogeneous composite and better fibre—matrix adhesion. In addition, industrial production of untreated hemp fibres cannot be done without damaging the fibres making the field retting the only alternative with much worse properties of 49 GPa and 0.162 for stiffness and porosity factor, respectively. The fungal retting is a simultaneous enzyme production and utilization treatment. Industrial introduction of fungal treatment could be possible in the form of solid state fermentation and would according to this study provide fibres of significantly higher quality than field retting. The maximum composite properties obtained with *P. radiata* Cel 26 were 307 MPa for strength and 35 GPa for stiffness corresponding to V_f_ = 51%. These properties were higher than obtained by Thygesen et al. (2007) with 196 MPa and 32 GPa, respectively,  which was lower due to the lower fibre content of 32%.

### Overall concluding perspectives

Field retting and *P. radiata* Cel 26 retting of hemp were assessed in hemp fibre/epoxy composites and compared with a pure enzymatic retting approach. It was found that the abundance of fungi decreased with extended field retting duration, while the abundance of bacteria increased. Field retted fibres exhibited much higher glucanase and lower polygalacturonase activities than *P. radiata* Cel 26 retted fibres. As a result, retting with *P. radiata* Cel 26 could degrade non-cellulosic components from hemp fibres at highest selectivity. Composites with *P. radiata* Cel 26 retted hemp fibres had significantly higher strength (307 MPa) than composites made with field retted hemp fibres (248 MPa). However a pure pectinase approach resulted in even higher composite strength (325 MPa) (Liu et al. 2016b). Despite this disadvantage the fungal treatment has the advantage of giving more pure cellulose and avoiding costs of enzymes for the process.

## Abbreviations

ABTS: 2,2'-azino-bis(3-ethylbenzothiazoline)-6-sulfonate
DNA: deoxyribonucleic acid
E_c_, E_f_, E_m_: stiffness of composite, fibres and matrix, respectively
ESEM: environmental scanning electron microscopy
HPAEC-PAD: high-performance anion-exchange chromatography with pulsed amperometric detection
ITS: internally transcribed spacer
rRNA: ribosomal ribonucleic acid
UTS: ultimate tensile strength
XG: xyloglucan
